# Practical Remarks on Near-Sight and Impaired Vision

**Published:** 1847-01-01

**Authors:** 


I. Practical Remarks on Near-Sight, Aged-Sight, and Impaired Vision. With Observations on the Use of Glasses and on Artificial Light. By *William White Cooper*. 8vo. pp. 216. Churchill, 1846.

II. A Manual of the Diseases of the Eye. By *S. Littel*, Junr. M.D. 2nd Edition, 8vo. pp. 360. Philadelphia, 1846.

III. Annales d'Oculistique. Tom. XIII.—XVI. Paris, 1845-6.

Among the inconveniencies of the practice we have so frequently censured of addressing the public and the profession simultaneously, is the pecuniary grievance, which, although slight in importance compared to the other evils, is yet worth notice. Why should the educated medical reader pay for the paper and print wasted in endeavouring to instruct the non-professional peruser in the A, B, C of medical science: and why is the latter to be mulcted for the information offered to the former, which can be of no service, or may prove prejudicial to himself? Even Mr. Cooper's little work illustrates this on a small scale; for in it we find an account of the laws of optics and of the anatomy and physiology of the eye, which any tyro would be ashamed to confess he stood in need of; and a description of the treatment of ophthalmia and incipient amaurosis which the non-professional reader could not comprehend, nor would be benefited by comprehending. As respects the professional reader, we do not find that Mr. Cooper supplies him with much information additional to that which was already accessible to him in works upon optics and ophthalmology, and we scarcely think the parcelling out small portions of large subjects into separate treatises, unless novel views or extensive series of facts are to be adduced, a useful practice. Still, the work is written in a lucid and interesting manner, and will be found useful to those who have not hitherto turned their attention to the important subject upon which he treats. And what more important matter can engage the attention of the practitioner than the *preservation of sight?* Yet the subject has been unduly neglected by the profession, and either left to Nature's uncertain ministrations, or consigned to the care of quacks, oculists, or opticians. So strongly has M. *Sichel* of Paris, felt this to be the case, that he has for some time past devoted a clinical lecture weekly to the consideration of all that relates to spectacles. Some of these are now publishing in the periodical whose title we have quoted at the head of this article, as a series of papers upon "*Spectacles and the pathological conditions consequent upon their injudicious employment.*" They are of an interesting and important character, and we proceed to lay before our readers the substance of some of them.

*Accommodation of the Eye to different Distances.*—The point of distinct vision, M. Sichel observes, varies much in different persons, as also does the space through which it continues in operation. The less an individual has been compelled by the nature of his occupations to constantly limit himself to one visual distance, and the more he has been in the habit of alternately exercising his eyes on near and distant objects, the more considerable will the extreme limits of his sight be. Those who have much varied the distances in this way can discern near objects as distinctly as the myop, and distant ones as well as the presbyop. The notable diminution or complete loss of the power of accommodation is the cause of the extreme conditions of myopia and presbyopia, and of the ill-consequences which follow the injurious use of spectacles.

*On Spectacles.*—Glasses of 48 inches focus are those of the lowest power ground for the presbyopic eye in London; and, until the last few years, the same was the case in Paris, when M. Sichel introduced those of 72, 80, and even 96 inch focus—the last being little else than a plane surface. The opticians at first ridiculed the employment of glasses of so low a power, but they have since admitted their great utility under various circumstances. Spectacles are often made oval and small, for the sake of neatness, but M. Sichel recommends their glasses being round and large, so as to cover not only the eye but its immediate vicinity. This is especially necessary for the coloured preservers intended to mitigate the effects of the impression of light in various affections of the eye; for otherwise the light is reflected at the sides, and the centre alone being protected, the very contrast increases the injurious effect. So, too, when even colourless glasses are employed, if they are small and oval, an irregularity of refraction takes place which may cause much confusion of vision and even diplopia. Care must be taken in fitting and wearing the spectacles, so as to prevent their falling forwards on the nose, and giving rise to a different degree of refraction. They should be as close to the eye as possible, but not so close as to allow the eyelashes or other appendages to touch the glasses. Upon this subject Mr. Cooper offers a useful caution.

"There is one point of considerable importance, which is seldom regarded, viz. the fitting of the spectacle frame, so that the centre of each glass shall be exactly opposite the pupil of the corresponding eye. There are scarcely two persons of precisely the same width between the eyes, and yet in the majority of cases this fact is entirely lost sight of in the selection of spectacles. A person finds that when, at an optician's, he looks through a lens of a certain power, it suits him exactly. He sees delightfully with it, and forthwith orders spectacles of that power. He tries them on as soon as he receives them, anticipating with eagerness the comfort they will afford him; instead of which he finds he can hardly see at all, or, if he does, his eyes soon feel fatigued. The glasses are right, the error is in the frame. Unless the width between the eyes is such, that the centre of each glass is exactly in front of the eye which it is to assist, the rays that pass through the lens will not all enter the pupil, and the spectacles will be comparatively valueless. Care should be taken then, in every case, to have the bridge made of such a curve, and such a width, that the position of the lenses as regards the eyes shall be perfect, both horizontally and vertically." P. 125.

Mr. Cooper also thus expresses his opinion upon the substitutes for spectacles.

"Some persons prefer to use an eye-glass, others reading-glasses, in lieu of spectacles. Reading-glasses, however, are objectionable, from their not being firmly fixed in front of the eyes. The motion of the head not being in accordance with that of the hand which holds the glasses, has the effect of trying the eyes exceedingly in their constant and ineffectual endeavour to adjust themselves to the position of the glasses, inducing unnecessary fatigue to the eyes, and rendering necessary an earlier resort to glasses of a higher power than would have been required had proper spectacles been adopted from the commencement.

"But a single eye-glass is more injurious still; and many young men, who, from shortness of sight, or a singular vanity, have thought proper to use a quizzing-glass, have had reason to regret it to the end of their lives. The consequences to perfect vision are serious, for as one eye is made to do more work than the other, an alteration in their relative strength takes place; the result is, that sooner or later, when the person resorts to spectacles, he finds that the lens which suits one eye will not at all suffice for the other. Watch-makers, and other artists, who work with a magnifier, are very subject to this imperfection of vision, and generally find that they see better with one eye than the other. If, instead of always applying the magnifying-glass to one eye, they were to use the other eye in turn, a habit which might easily be acquired in early life, although with difficulty afterwards, they would preserve the power of their eyes more equally, and the perfection of vision longer; for, by using the eyes alternately, rest, and an opportunity of recovering from the fatigue produced by the exertion of looking through the magnifier, would be afforded to each. In like manner, those who indulge in microscopical or astronomical pursuits, should learn to use either eye indifferently, instead of always trusting to one, although we almost instinctively apply the right eye to a telescope or microscope." P. 118.

*Presbyopia—Far-sightedness—Aged-sight.*

Although the name of this condition of the eye is (πρεσβυς, old), significative of the fact of its usually forming one of the first indications of the approach of age, it is by no means confined to any period of life—many eyes being originally presbyopic as others are myopic. We need not give the general description of the symptoms of the condition as furnished by M. Sichel and Mr. Cooper, nor the marks distinguishing it from cataract and amaurosis set down by the latter; merely observing, in respect to these last, that we are surprised Mr. Cooper dwells upon the diagnostic signs derived from general symptoms, when so simple a one as the trial of the effect of a convex lens would, in the majority of cases, dissipate all doubts. M. Sichel has some valuable observations upon this affection. He dwells much upon the *hygienic precautions necessary* to be observed by the presbyop. Thus, when engaged in writing, reading, or minute occupations of any kind, he should always *place the object as distantly as possible from his eyes,* consistently with distinct vision. Having by experiment ascertained what this point really is, he should in future, during all assiduous labours, carefully observe it. In less trying occupations he may exercise his eyes at less distances, so as to accustom them to perceive within their normal focus. In both cases, he must from time to time *interrupt his labours, and direct his eyes to the most distant objects within the sphere of his vision.* After five or six mimutes' intense exertion of the eyes on minute objects, he should cast them towards the walls of the apartment or out of the window. An interruption of this kind of only a few seconds preserves the power of accommodation of the eye, and prevents the enfeeblement of the sight; and in this way M. Sichel has repeatedly seen a commencing amblyopia dissipated. The interruption is very slight, and by habit becomes almost imperceptible. The more closely these directions are observed, the longer will the eyes be preserved without the necessity of resorting to glasses; and the minuter the occupation the more necessary are the precautions. If possible, people who are presbyopic should not choose occupations necessitating an exertion of the eyes on minute objects; and many dress-makers, shoe-makers, tailors, &c., would do much better as domestic servants. M. Sichel particularly alludes to the injury which is inflicted upon the eyes of *children* by allowing them to bend over their tasks, so as to bring their eyes close to the written characters, instead of obliging them to hold themselves erect, taking care that the letters of the words are sufficiently large for them.

*Degree of Light required in Presbyopia.*—For the purposes of exact vision, and especially for near objects, the presbyopic eye requires a bright light, so that half-lit apartments are very injurious to it. Lamps furnish a much better and more equable light than candles. Coloured glasses, which have been erroneously termed "*preservers,*" should only be employed when the eye is temporarily exposed to the reflection of some exceedingly vivid light. In other circumstances they are very hurtful, by accustoming the eye to artificial darkness, and covering everything with a blackish tint, which prevents objects being accurately distinguished without effort. In this way, amblyopia and photophobia may be induced; and many such cases have been promptly relieved by the prompt removal of the patient from an obscurity to which he had been erroneously consigned. When exposed to very bright reflection, glasses of a light greyish-blue are the most suitable.

*Glasses in Presbyopia.*—It is usually about 40 that the presbyopic eye requires the aid of spectacles. While the sight for distant objects may be even improved, that for near ones, especially if not well lighted, or removed to a distance, becomes impaired. The fixing the eye is likewise painful and fatiguing, and if the irritation go on, it may lead to amblyopia. Before symptoms reach this point we must have resort to glasses, employing them at first only during artificial light or minute occupations. The rules before-mentioned are still to be observed, and especially that of holding objects as distinctly as possible from the eyes consistently with distinct vision. In respect to the number of the glass which should be commenced with, it is an error to suppose that this is always the same for the same age, and opticians generally recommend a far too high power, and increase this far too suddenly. Even excellent writers upon affections of the eyes fall into the same error; and thus Mackenzie recommends a 36-inch focus for persons of 40 years of age, and a 30 for those of 45. As the result of numerous experiments, M. Sichel commences with a 72-inch, which in general answers very well for persons who have not yet employed glasses, and who have not delayed resorting to them too long. Frequently, however, and especially in persons younger than 40, 80-inch or 96-inch answer excellently for a long period. In trying spectacles we should be able to discover objects distinctly (but not magnified) at a little less distance than we see them imperfectly without them. All glasses, more or less, destroy the accommodating power of the eye, and that in proportion as they are strong; so that we must be content with a power just sufficient, increase it only by almost insensible gradations, and from time to time dispense with it and fix the eye on some distant object, and even occasionally employ the unassisted eye for a short time on minute objects held at a distance. In the earliest transitions of size the actual differences are very slight, as from 96 to 84 and 72-inch; but, as we descend, these become more marked, and to prevent their being too abrupt, M. S. employs 66 and 54-inch, recommending the patient to retain each glass as long as possible, and decrease it only six inches at a time. It is an error to say that a change of glasses is *always* necessary. It often becomes so only because too high numbers have been began with, and cautions neglected: so that, when we hear a patient complain of his glasses being ineffectual, we must examine whether he has not originally used too powerful ones, and thus induced amblyopia. By employing this slowly graduated scale, M. S. rarely finds a lower number than 48 required; while, when 48 is began with, a lower one than 36 never succeeds. In very young persons, or where amblyopia complicates the presbyopia, we should never commence with lower than 96, and keep at 80 as long as possible.

*On Amblyopia (Defective Vision) produced in Presbyopia by the too*

*assiduous Employment of the Naked Eye.*—If a presbyop continues to employ his eyes injudiciously, a condition very difficult to distinguish from incipient amaurosis may be produced. This is *presbyopic amblyopia,* or amblyopia resulting from presbyopia. The visual disturbance is very great, especially with artificial light or in obscure places, and is characterized by its disappearance under the use of convex glasses. If neglected, an asthenic form of amaurosis results. This species of amblyopia is especially found in engravers, miniature-painters, watch-makers, literary men, dress-makers, &c.; and more particularly in those persons who, having lived in the country, and been accustomed to long sight, afterwards have their eyes suddenly employed on minute objects. It is likewise especially found in those in whom a constitutional debility of the retina is conjoined to presbyopia. In presbyopia, amblyopia is generally the effect of excessive exertion of the eye, while in myopia it usually results from constitutional causes, such as cerebro-ocular congestion, abdominal plethora, &c. This condition has been described by various authors under different names (by Mackenzie, as Asthænopia or weakness of sight), but its true nature has not been appreciated by them, in consequence of the presbyopia having been overlooked.

In treating this affection, M. Sichel recommends total or partial abstinence from minute employment, or, at all events, its interruption from every two to ten minutes, according to the gravity of the case, in order that the eyes may be directed to distant objects—applying to them also spirituous, or, if the case is old, stimulating lotions. In this last circumstance, too, blistering the brow and temples, and the use of convex glasses, are indicated. Even when amaurosis has commenced, these measures will usually triumph over it, employing in this case therapeutical means more energetically and glasses more sparingly. As to these last, when the eyes have been sufficiently rested from employment and amelioration has commenced, we may allow the patient several times a day to employ, for a few minutes, glasses strong enough to admit of reading without any fatigue, or without the necessity of looking too close. As the case improves, their refractive power may be diminished.

We may here cite a case of *temporary presbyopia* quoted by Mr. Cooper.

"On the 17th of April 1840, a boy, eleven years of age, was brought to Edinburgh from the country for the opinion of the late Dr. James Hunter. Fifteen days previously he was at school in perfect health, when one evening the discovery was made that he could not read common type, nor distinguish accurately any very small or near object. There was neither pain or symptom of disease in either eye, but the vision of each was equally affected. The general health of the lad was unimpaired, and he had not received any injury either of the eyes or any other part. During the two following days the sight became worse, but after that it had remained stationary. Excepting the administration of some purgatives no treatment had been adopted. Previous to the attack his sight had been extremely good, and he had not been troubled with worms or other ailment since infancy. The eyes, upon examination, appeared perfectly healthy, and the only complaint made was the inability to read common print, or to see minute or near objects: distant objects, he thought, were as distinct as ever (although it was subsequently ascertained that his distant vision was slightly affected). Large type was best seen at 11 inches from the eye; small print could not be read at all; distant objects were ascertained pretty accurately; and the power of the two eyes seemed equal. Concave glasses rendered his sight much less distinct, convex glasses improved it much. Dr. Hunter strictly prohibited the use of spectacles, and prescribed a combination of anthelmintic and tonic treatment, with spare diet and plenty of exercise in the open air. No worms made their appearance. The presbyopia continued until the end of May, when an amendment was perceived which increased daily, and in about ten days the sight was perfectly restored." P. 89.

*The Employment of Glasses in Amaurosis.*—Charlatans have long been in the habit of employing convex glasses in amaurosis, and those practitioners who have followed their example have explained the occasional benefit thence resulting by the excitement of an asthænic retina; but, according to M. Sichel's views, they are only useful when the amaurosis has followed presbyopia. In these cases, even, very convex glasses should not be employed before fully trying the effect of feebler ones. He has long been aware of the modifications which the difference in the length of normal vision impresses on the symptoms. Thus, in the condition of amblyopia from presbyopia, slightly voluminous objects are best seen when held at more or less distance—the sight at a distance remaining good although impaired for near objects. In such cases, sight is notably improved by convex glasses. After a long period, however, when the patient's sight becomes shortened to a great extent, glasses are of no use, and the amaurosis is incurable. In myopia the sight, while enfeebling also at a far earlier period, becomes shorter, and convex glasses do not facilitate vision, unless placed very near the eye so as to act as magnifying-glasses, at the same time stimulating, and eventually injuring the retina. Thus, we would expect, as in fact is the case, that, in amaurosis from presbyopia, glasses would prove useful, and the more so as its origin is local—*i. e.* produced by fatigue of the organ of vision. Hence they frequently prove of service both in the treatment and the diagnosis of the affection. Strong glasses must not be employed if the case is not a very old one, the presbyopia excessive, the foci of the eyes unequal, or the patient unable to suspend his occupations. It is doubtful if any case of cure of myopia by these means has ever occurred.

There is a variety of *presbyotic amblyopia*, however, from which patients derive no benefit from glasses, and on enquiry such persons will have been found to have employed their unprotected eyes long after defective vision had manifested itself. The eye has thus been forced to accommodate itself to minute objects until positively unable to continue to do so. In this case the retina must be allowed to recover itself by repose, and by frequent exercise on large and distant objects, after which the 96-inch focus may be employed. Often, however, stronger glasses will be required, the patient by exertion of the unassisted eye having contracted an *acquired myopia.* Although this form of amblyopia is usually of slow occurrence, it occasionally comes on rapidly, if the eyes are sedulously employed upon smaller objects than they are accustomed to. This is the case with some children who are too early employed in reading, sewing, &c. Presbyopic amblyopia generally occurs without complications, but it may exist in conjunction with cerebro-ocular congestion, dysmenorrhœa, constipation, &c. — conditions which much favour the occurrence of amblyopia in presbyopia. These complications must be removed before recurrence is had to glasses or local stimuli. It may also be associated with various other diseased conditions of the eye, often rendering their diagnosis very obscure. The complication of presbyopia with conjunctivitis has frequently given rise to the supposition of an amblyopia which has had no existence in fact.

*Some of the Consequences of Presbyopia.*—Among these M. Sichel mentions (1) *Ocular Neuralgia* as of frequent occurrence. At first occurring only after long exertion of the eyes, it eventually comes on as soon as this is commenced. Any exertion whatever indeed of the eye induces pain, and this may occur when it is quite at rest; and the affection may become complicated with rheumatism or cerebro-ocular congestion. At an early stage, rest and appropriate glasses suffice for the treatment. Later, absolute rest is essential, opiates being given internally or veratria applied to the brow—the complications being properly regarded. But all measures are useless unless first absolute rest, and then only the gradual use of the eye, be observed. Both eyes are usually affected, and, when this is not the case, we should examine them and see whether the affection do not arise from their foci being unequal. In rare instances it is induced by the use of strong glasses.

(2), *Mydriodapsia, or Muscæ Volitantes.*—These almost always arise from a rash use of the organ. In presbyopia they are found when the naked eye is used, or when one eye is fatigued by an inequality of focus. Common as is this affection from the abuse of glasses in myopia, it is rarely produced by the use of convex glasses in presbyopia. The muscæ volitantes must, in regard to prognosis, be carefully distinguished from the fixed darkened spot called *scotoma*, the sure forerunner of amaurosis.

(3), *On acquired Myopia, or Presbyopia converted into Myopia.*—As the presbyop can only apply his eye in working by forcibly subjecting it to a too short focal distance, we can easily see how assiduous labour under these circumstances may eventually destroy the accommodating power of the eye for the observation of distant objects, and so shorten the focal distance as to induce a *secondary myopia*. The continuance of such employment tends only to increase it, to which other circumstances also sometimes contribute. Thus, in ignorance of the determining cause, to relieve his uneasiness during the employment of the eye, the person may be recommended to use a concave glass, which only aggravates his condition. We may observe acquired myopia in its *simple form;* unaccompanied by any weakness of vision—the person having become myopic from the mere exertion of his eyes. This affection comes on slowly, and is rather found in persons who have acquired the bad habit of placing objects too near their eyes, especially by stooping at their work, than in those who have too much employed them. The majority of myopiæ are so induced. In this simple form the organ of vision is perfect, providing the object be placed at an appropriate distance. When it is not of long-standing, it suffices to gradually increase the distance of the objects from the eyes, and exercise these upon distant ones at frequently-recurring intervals. The use of concave glasses is to be rigorously prohibited; nor should convex ones be employed when there is no complication of amblyopia, the original presbyopia is not excessive, or the individual still young. This variety of myopia offers an explanation of the curious phenomenon mentioned by most authors as occasionally occurring, the *spontaneous return of the power of working without the use of convex glasses.* But the acquired myopia may be *complicated with amblyopia.* The amblyopia in these cases is generally asthænic, although it is sometimes attended with cerebro-ocular congestion. The treatment, as in all cases wherein amblyopia is present, is difficult, and there is always danger of the affection becoming converted into permanent amaurosis. Absolute rest from occupations, with exercise of the eye on long distances in the open air, are indicated—the accompanying congestion or other diseased conditions being removed by appropriate means. All glasses must be interdicted. The amblyopia may in this way be removed even when approaching to amaurosis; but the myopia remains, diminished however, in proportion to the docility and the perseverance of the patient. At this period, the cautious use of convex glasses may be allowed during a portion of the day.

We may quote some of Mr. Cooper's remarks upon the *Preservation of the Eyes.*

"Those whom circumstances compel to study in the evening, should select that kind of work which is least distressing to the eyes. They should especially avoid indistinct writing or small print. The 'Diamond Editions,' in which the print is extremely small, are very hurtful to the eyes. I have a volume of Burns' Poems thus printed, and if I attempt to read it, my eyes feel strained, and the appearance of muscæ volitantes is excited before I have perused half a page. Reading by fire-light, or simply gazing at the fire, when sitting alone, or in a contemplative mood, is highly injurious to feeble eyes, and should be avoided by all. It is not advisable to read by twilight: too little light is as pernicious as too much, yet many persons will, evening after evening, try their eyes in this way rather than burn a candle. It is injurious to the eyes to be long exposed to the reflection of a strong light, whether artificial or natural, such as the reflected sun-shine from the page of a book. * * * * * * * * * In reading and writing, just that amount and quantity of light, whether natural or artificial, should be allowed, which, while it thoroughly illuminates the object, feels grateful and pleasant to the eyes. This desideratum can never be obtained without due regard to the position of the light. The light cast upon a book while the candle is in front, is by no means pleasant, and the glare of the flame is very trying to weak eyes. It will be found that, if the candle or lamp be placed behind the reader, a little elevated and slightly on one side, the pleasantest and least injurious effect is produced; for the light then reflected to the eyes is less distressing, and at the same time the eyes are perfectly protected from the heat and glare of the flame. It would be well if in our public buildings more attention was paid to the position of the lights: it is very distressing to sit in a gallery immediately opposite the glare of a gas-burner or lamp, for an hour or more; the eyes frequently do not recover from the irritation thus excited for several days. Not only might the evil be easily removed by employing lights of greater power and placed nearer the ceiling, but there would be a great advantage gained from the increased purity of the air. * * * * * * * * It cannot be too strongly urged upon any one about to use spectacles for the first time, that that power which will enable him to read without much exertion by candle-light, is the only power suitable for him. It is by candle-light only he should use glasses at first, and so soon as he finds that he stands in need of glasses by day as well as by candle-light, and the glasses he uses no longer afford him sufficient assistance by candle-light, it will be proper to use the next power for the evening, but the evening only, and to allow himself the use of the others—and their use only—during the day. The greatest caution as to increasing the power of glasses should be observed, for persons who change their glasses unnecessarily, increasing the power each time, are exhausting the resources of art instead of economising them as much as possible. Optical aid can only be extended to a certain point, and the steps to that point should be as slow and as numerous as possible. By exercising prudent precautions, persons may often attain great age, and yet never require the aid of glasses beyond a very moderate power: others, on the contrary, who from ignorance frequently increase the power of their glasses, may run through the whole assortment, and leave themselves only the most inconvenient resources to fall back upon—viz. the very highest powers." Pp. 100-107.

This is very sound advice, and, if more generally followed, would prevent much disappointment and vexation. It is precisely in accordance with that so emphatically reiterated by M. Sichel, but Mr. Cooper recommends a power (48 inches focus) which the former practitioner regards as far too high to commence with, and an augmentation of it, which he looks upon as not sufficiently gradual. We think the low powers recommended by M. Sichel from their conservative tendency are well worthy a trial. They are to be found in the optician's shops of Paris, and would soon be procurable in London if sought for.

*Myopia—Shortsightedness—Near-sight.*—This subject will not occupy us long, as M. Sichel's observations upon it have not yet been published, and those of Mr. Cooper do not possess much novelty. He describes two varieties of the affection—1, that resulting from a too convex state of the cornea or lens, the too great distance between the cornea and the retina, or an undue density of the media. Any of these causes unduly increase the refracting power of the organ, and bring the rays to a focus before they can reach the retina. 2. Myopia resulting from the loss of the adjusting power of the eye.

Myopia from structural peculiarity is often discovered only by accident, a person casually observing the improved vision which is produced by his looking through a concave glass. Short-sighted persons also see better by holding objects very near their eyes, in consequence of the divergency of the rays which is thus produced, and the greater distance backwards at which the image is formed.

"Distant objects appear large to near-sighted persons, because a distinct picture is formed only at the point of intersection of the rays proceeding from an object, and as this point falls short of the retina in these persons, the retina receives the rays beyond the point of intersection, and consequently where they are more extended. Near-sighted individuals often write a very small hand: the proximity of the letters to the eyes increasing the visual angle subtended by them, and causing them to appear distinct enough. If a near-sighted person looks through a pin-hole in a card, he can distinguish objects clearly at a greater distance than before; this is effected by excluding the circumferential rays, which by their too speedy conveyance, would tend to form foci *before* they reach the retina, and thus cause indistinctness of vision. The pupils of the eyes of myopic persons are generally large, and their habit of half closing the lids when looking at distant objects is upon the same principle, that is, for the purpose of excluding all but the central and direct rays." P. 45.

Myopia may result from disease, as *Hydrophthalmia,* which is especially liable to occur in strumous children from inflammation of the cornea or of the aqueous membrane. After the acute stage has subsided, the anterior chamber is observed enormously enlarged, the cornea protuberant, and the iris carried backwards and apparently concave. "In some cases there does not appear to be much alteration in the form of the cornea; it appears thinner, but does not assume the pointed form characteristic of conical cornea. The extra prominence is general." There is more affection of vision than the altered form of the cornea will explain, the retina being usually more or less impaired in its functions. The early stage of *Conical Cornea* may be mistaken for a mere myopic condition of the eye; and Mr. Cooper has met with a case of *Congenital Cataract* that was for many years treated as aggravated myopia.

Mr. Cooper cautions the myopic patient not to increase the power of his concave spectacles too rapidly, or to wear them constantly. He quotes Dr. Kitchener's excellent remarks upon this subject; and those of our readers who have not seen this eccentric but clever author's work, "The Economy of the Eyes," will do well to peruse it, containing as it does much valuable information, interlarded though this be with all kinds of extraneous observations. One or two of Mr. Cooper's cautionary remarks may be quoted.

"Dr. Wells, in his Experiments and Observations (London, 1818), states that he was informed by Mr. George Adams, an eminent optician, 'that he does not know a short-sighted person who has had occasion to increase the depth of his glasses if he began to use them in the form of spectacles; whereas, he can recollect several instances where those have been obliged to change their concave glasses repeatedly for those of higher powers who had been accustomed to apply them to one eye only.'

"The above is a fact which ought to be more generally known, and is an argument against the use of single eye-glasses. Near-sighted persons are very apt to stoop while at their studies. To avoid a practice so injurious to the figure and the health, a high desk should be provided to read and write at, and such glasses should be used as are just sufficient to enable the parties to pursue their occupation at the ordinary reading distance, that of about 14 inches. In all cases of myopia, and especially in early life, or when the affection is just commencing, it is highly important that any tendency to an over-supply to the eyes should be counteracted by a proper quantity of bodily exercise. The adjusting powers of the eyes should also be daily exercised in the attempt to obtain a distinct view of objects at a distance." P. 59.

Of the variety of *Myopia dependent upon the Loss of the Adjusting Power of the Eye*, much has been already said when treating of Presbyopia. The eyes, when excessively exerted upon minute or near objects, become unable to adjust themselves for the due perception of the more distant ones. Mr. Cooper remarks the prevalence of concave spectacles among the Germans, who are such hard students, and Mr. Ware found of 127 students at one of the Colleges at Oxford, 32 employed an eye-glass or spectacles. Mr. Cooper relates a few cases in which the employment of the eye upon minute objects gave rise to the production of a myopia, which was cured by rest of the organ, its exercise on distant objects, country air, and the abstaining from, or the cautious use of, glasses.

*Impaired Vision.*—Hard reading, and a variety of occupations among the working-classes, give rise to this affection, the prevalence of which among the latter the author says may be judged of by the fact of no less than 329 of such cases having been relieved between Jan. 1842 and Jan. 1846, at the North London Ophthalmic Institution—a conclusion, however, not easily drawn in the absence of a statement of the total number of patients attending during this period.

In the *treatment* of this affection, rest of the eyes, at the least for a while, is of the first importance; and those persons whose occupations forbid this, should at all events intermit them repeatedly and apply cold water to the eyes, working as little by artificial light as possible. If there is marked congestion of the retina, as shewn by brilliant spectra, photophobia and deep-seated pain, cupping from the temple is indicated. Successive blisters to the brow are very useful also; or the following rubefacient may be applied upon a piece of flannel to the forehead for two or three minutes every, or every other, night: ℞. *Liquor. ammon. fort.* ℥ss., *Sp. rorism.*, *Sp. camph.* āā ℥j., *Sp. v. r.* ℥ss. M. The bowels are to be kept duly regulated, and an alterative course of mercury often proves of great service in arresting disease in the retina. When general debility is present, tonics, in combination with counter-irritation, and especially bark and iron, are the appropriate medicines, duly regulating the bowels by means of aloes and myrrh, and carefully watching the condition of the various secretions. In these subjects, cold ablution, followed by active friction with a rough salted towel and a horse-hair or Indian flesh-glove, is a very invigorating procedure. When there is weight or oppression of the head the shower-bath should be employed, highly sensitive persons even well bearing the shock it occasions if they stand the while in hot water. The due regulation of the diet, and the removal of disorders of the digestive organs, in such who suffer from them, are of great importance.

*Artificial Light.*—Every one is aware of the great superiority of daylight over any kind of artificial light. Here are some of the reasons for this.

"When the eye is exposed to light in which the red and yellow rays prevail, the colours in excess produce, first an excitement, and afterwards a degree of debility of the retina. Consequently that light which approaches the nearest to white is best suited to the eyes, and that which partakes most of red, the worst. Another cause of the injurious effects of artificial light is the direct and concentrated manner in which it acts upon the eyes. The rays from a candle or lamp fall direct upon the object which a person is regarding (the page of a book or sheet of writing-paper for instance), and are thence reflected into the eyes, carrying with them a considerable quantity of heat, which irritates and inflames the external coats of the eyes, and the lining membranes of the lids. A great portion of the heat which accompanies the sun's rays is absorbed during the repeated reflections from the atmosphere and clouds, or from the surface of the earth, which the light undergoes before it reaches the eyes. Another cause of the distress produced by artificial light upon some eyes, is the fact of the rays not falling in parallel but divergent lines upon the object, from which they are reflected in equally divergent lines; consequently, indistinctness of vision results from the want of definition of the object: whereas the rays of the sun, owing to its immense distance from the earth, may be regarded as parallel. The unsteadiness of artificial light is another serious evil to persons suffering from weak eyes. One great superiority of day-light over artificial light is its perfect evenness. It is some inequality, either in the current of air, or in the supply of combustible material, that renders the common flame unsteady and varying." P. 167.

Among the various kinds of artificial light, Mr. Cooper disapproves of that from *gas*, owing to its yellow colour and heating power, these rendering it injurious to the eyes when employed upon minute objects. It evolves carbonic acid also in large quantities, and some of the gas escapes unconsumed; so that it should never be employed in apartments unless some special means for their ventilation is also in operation. The *camphine* lamp burns with a pure flame possessed of great power of illumination; but it should not be brought too near the book, or ordinary candle-light will in future be found insufficient. "By the injudicious employment of too brilliant a light, for the purpose of study, the sensibility of the retina may be blunted by slow and imperceptible degrees, without the patient being alarmed by any sudden impairment of vision, or marked difference in his sight." The light of a well-constructed Argand Lamp is of a very superior description. Mr. Cooper recommends *wax candles* for those who employ their eyes by night, as affording sufficient illumination without fatiguing or heating the organs. "I find, from experience, that the light they afford enables me to write longer with less distress to the eyes, less irritation of the lids, and a greater amount of general comfort than any other." Composition candles formed of stearine and wax, also give an excellent light, and are less expensive. As artificial light proves injurious from an excess of the red and yellow rays, much of its inconvenience may be obviated by surrounding it with a shade coloured blue in its inner side, which reflects a whiter or purer light. The same object may be attained by using a pale blue glass chimney for the lamp, or allowing the light to pass through a bluish fluid. Contrivances of this kind are to be preferred to wearing shades or spectacles.

Although, as we have stated, we do not approve of the system of morselling an entire subject, the account of Mr. Cooper's work we have given shows it to be one of considerable merit.

Dr. Littel's "Manual" will be found useful to those who are too much engaged, or, as is too often the case with the degenerate students of the present day, too indolent to consult more elaborate works. The descriptions are faithful, and the directions for treatment judicious. Their conciseness, however, and our want of space, prevent our giving a more detailed notice of its contents. We may remark, however, that Dr. Littel disposes of the operation for Strabismus in too off-hand a manner for our taste. That its efficacy has been ridiculously and mischievously exaggerated, and that sometimes from the most sordid motives, we admit: but, well-knowing the advantageous issue which has attended certain carefully-selected cases, we should indeed feel sorry in joining Dr. Littel in absolutely proscribing so simple a remedy for so grievous a deformity.

In our next number, we shall probably enter at considerable length into the subject of Diseases of the Eye, as M. Desmarres' and Mr. Wharton Jones' new works upon Ophthalmic Medicine will then be in our hands.

